# Antimicrobial peptide-like genes in *Nasonia vitripennis*: a genomic perspective

**DOI:** 10.1186/1471-2164-11-187

**Published:** 2010-03-19

**Authors:** Caihuan Tian, Bin Gao, Qi Fang, Gongyin Ye, Shunyi Zhu

**Affiliations:** 1 Group of Animal Innate Immunity, State Key Laboratory of Integrated Management of Pest Insects & Rodents, Institute of Zoology, Chinese Academy of Sciences, Beijing 100101, PR China; 2 State Key Laboratory of Rice Biology, Institute of Insect Sciences, College of Agriculture and Biotechnology, Zhejiang University, Hangzhou 310029, PR China

## Abstract

**Background:**

Antimicrobial peptides (AMPs) are an essential component of innate immunity which can rapidly respond to diverse microbial pathogens. Insects, as a rich source of AMPs, attract great attention of scientists in both understanding of the basic biology of the immune system and searching molecular templates for anti-infective drug design. Despite a large number of AMPs have been identified from different insect species, little information in terms of these peptides is available from parasitic insects.

**Results:**

By using integrated computational approaches to systemically mining the Hymenopteran parasitic wasp *Nasonia vitripennis *genome, we establish the first AMP repertoire whose members exhibit extensive sequence and structural diversity and can be distinguished into multiple molecular types, including insect and fungal defensin-like peptides (DLPs) with the cysteine-stabilized α-helical and β-sheet (CSαβ) fold; Pro- or Gly-rich abaecins and hymenoptaecins; horseshoe crab tachystatin-type AMPs with the inhibitor cystine knot (ICK) fold; and a linear α-helical peptide. Inducible expression pattern of seven *N. vitripennis *AMP genes were verified, and two representative peptides were synthesized and functionally identified to be antibacterial. In comparison with *Apis mellifera *(Hymenoptera) and several non-Hymenopteran model insects, *N. vitripennis *has evolved a complex antimicrobial immune system with more genes and larger protein precursors. Three classical strategies that are likely responsible for the complexity increase have been recognized: *1*) Gene duplication; *2*) Exon duplication; and *3*) Exon-shuffling.

**Conclusion:**

The present study established the *N. vitripennis *peptidome associated with antimicrobial immunity by using a combined computational and experimental strategy. As the first AMP repertoire of a parasitic wasp, our results offer a basic platform for further studying the immunological and evolutionary significances of these newly discovered AMP-like genes in this class of insects.

## Background

AMPs constitute essential components of innate immunity to rapidly respond to diverse microbial pathogens [1,2]. As key effectors, AMPs directly kill invaders by acting either as pore-formers or metabolic inhibitors [3]. Most AMPs are cationic polypeptides, usually smaller than 100 amino acids in length, with enormous sequence diversity. Based on structural characteristics, these molecules are roughly divided into three different groups [4]: 1) Linear peptides free of cysteines, often forming an α-helical conformation with amphiphilic surface, such as insect cecropins and mellitins, amphibian magainins and arachnidian meucins [5,6]; 2) Cysteine-rich peptides. The most representative of this group are insect-derived defensins, which belong to the CSαβ polypeptide superfamily and are conserved across the kingdoms of eukaryotes [7,8], even in bacteria [9,10]; 3) Peptides with unusual bias in certain amino acids, such as glycine-rich tenecin3 [11], proline-rich apidaecin [12], histidine-rich histatin [13] and tryptophan-rich indolicidin [14]. AMPs in this group are unstructured and often enter into bacterial cells to inhibit different metabolic targets [3,15].

Insects, as a rich source of AMPs, attract great attention of scientists in both understanding the basic biology of immune system and searching molecular templates for anti-infective drug design. So far, about 200 such peptides have been identified from insects. In *Drosophila melanogaster*, there are 20 AMPs characterized [16]. In the past ten years, a number of genome sequencing projects have promoted application of computational approaches for the discovery of components involved in innate immunity of a given species. Some examples include *D. melanogaster, Anopheles gambiae, A. mellifera, Tribolium castaneum *and *Bombyx mori *[17-21]. This will undoubtedly extend our knowledge in basic biology of insect innate immunity system. Furthermore, these data also provide new clues for elucidation of immunological adaptation of insects to environmental changes. For example, analysis of the complete immune system of *A. gambiae *results in the discovery of a marked deficit of orthologues due to gene loss and excessive expansion of some specific genes, which is significantly different from that of *D. melanogaster*. This possibly reflects differential selective pressures from different pathogens between these two species. These results also facilitate further in-depth analysis of the mosquito immune system's impact on the malaria parasite [17,22].

Parasitoids (Hymenoptera, Insecta) are a group of parasitic insects. Some of them attach significant vectors of human disease, such as house flies, roaches and ticks, and some of them are extremely important regulators of agricultural pests [23]. To study molecular mechanism of the innate immunity of parasitic wasps, the basic biology of their AMPs is needed to be established. As the first parasitic hymenopteran insect to have its genome sequenced [24], *N. vitripennis *provides a new resource for the identification of AMP genes in this class of insects in a genomic scale [25,26]. Here, we report systemic characterization of the AMP repertoire from the *N. vitripennis *genome, which provide a perspective for the understanding of a possible relationship between immunity and parasitism. In comparison with *A. mellifera *and several non-Hymenopteran insects, *N. vitripennis *has developed a more complex antimicrobial immune system through genetic duplication and exon-shuffling.

## Results

### Computational identification of AMPs in the *N. Vitripennis* genome

To identify *N. vitripennis *AMP repertoire, we employed three complementary approaches to database search of the *N. vitripennis *genome (see additional file 1). Firstly, we chose *A. mellifera *AMPs (e.g. mellitin, apidaecin, apisimin, abaecin, hymenoptaecin, defensins) as queries to recognize their orthologues in *N. vitripennis *by BLASTP and TBLASTN. Secondly, we performed pattern search by the Scanprosite program based on the cysteine arrangement pattern of CSαβ-type defensins [8]. This strategy allowed identifying a large family of DLPs with a typical CXXXC and CXC motif and an amino-terminal signal peptide, which can be divided into three distinct subfamilies, including the known navidefensins [25] and two new subfamilies named nasonins and navitricins, respectively. Thirdly, we screened putative secreted peptides of < 150 amino acids from the *N. vitripennis *proteome by recognizing an amino-terminal signal sequence, from which AMP-like peptides rich in specific amino acids were identified. All AMPs found by the above approaches were again used as query to carry out BLASTP and TBLASTN iteratively until no new hits appeared. As a result, we identified a total of 44 AMPs in the genome of *N. vitripennis*, as shown in additional file 2, in which only nahymenoptaecin-1 and five defensins are recently reported [25,26].

All these newly discovered peptides display remarkable AMP characteristics, as reflected by their small size and net positive charges at pH 7.0. Most peptides described here are smaller than 150 amino acids in length, except for nahymenoptaecin-1, nahymenoptaecin-2, nasonin-2 and nasonin-6, which all have undergone internal duplication (see additional file 2). *N. vitripennis *AMPs exhibit extensive sequence and structural diversity and can be distinguished into multiple molecular types including insect and fungal DLPs with the CSαβ fold; Pro- or Gly-rich abaecins and hymenoptaecins; horseshoe crab tachystatin-type AMPs with the ICK fold; and a linear α-helical peptide.

To confirm the reliability of our computational prediction, we undertook the *N. vitripennis *EST database search and found more than 60% peptides have corresponding transcripts, suggesting the genes encoding these peptides are expressed at the transcriptional level. For remaining 40% peptides whose cDNA sequences were not found in the EST database, one possibility is that their expression depends upon suitable microbial challenges [27] or is associated with different developmental stages of *N. vitripennis*, as observed in the *D. melanogaster *antifungal drosomycin [28], although pseudogene or wrong prediction resulted from computational methods cannot be completely excluded.

### Classification of *N. Vitripennis* AMPs

#### Defensin-like peptides

##### Classical insect-type defensins (CITDs)

Defensins with the CSαβ structure are crucial effectors of innate immunity [7] which have been found in many insect species, the majority of them coming from different orders of the subclass Neoptera (Diptera, Coleoptera, Lepidopera, Hemiptera and Hymenoptera). These AMPs are primarily active against Gram-positive bacteria likely by forming voltage-dependent channels in bacterial membrane leading to a loss of cytoplasmic potassium [29,30]. Their protective roles have been well documented by *in vivo *targeted disruption of *defensin *gene which resulted in the death of *A. gambiae *after Gram-positive bacterial infection [31]. Insect defensins consist of 30-50 amino acids linked by three or four disulfide bridges. They share a common CSαβ structural motif with some functionally related scorpion neurotoxins targeting various ion channels, protease inhibitors, and even a plant sweet taste peptide [8,32].

**Figure 1:**
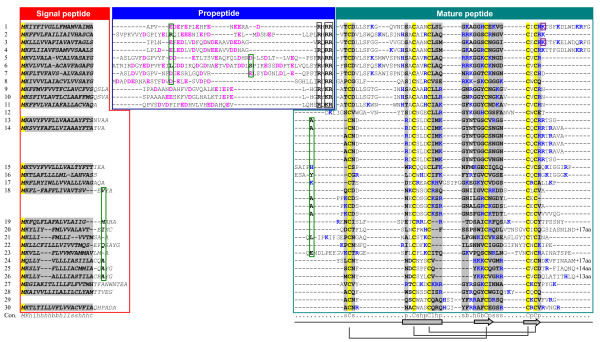
**Multiple sequence alignment of navidefensins, nasonins and related peptides**. 1: Amdefensin1(Am); 2: Amdefensin2(Am); 3: Navidefensin1-1(Nv); 4: Navidefensin1-2(Nv); 5: Navidefensin2-1(Nv); 6: Navidefensin2-2(Nv); 7: Navidefensin2-3(Nv); 8: Navidefensin3(Nv); 9: Phormicin(Pt); 10: Sapecin(Sp); 11: Dmdefensin(Dm); 12: Heliomicin(Hv); 13: Nasonin-1(Nv); 14: Nasonin-2(Nv); 15: Nasonin-3(Nv); 16: Nasonin-4(Nv); 17: Nasonin-5(Nv); 18: Nasonin-6(Nv); 19: Nasonin-7(Nv); 20: Nasonin-8(Nv); 21: Nasonin-9(Nv); 22: Nasonin-10(Nv); 23: Nasonin-11(Nv); 24: Nasonin-12(Nv); 25: Nasonin-13(Nv); 26: Nasonin-14(Nv); 27: Cobatoxin-1(Cn); 28: Cll-Defensin1(Cll); 29: Ps-Termicin(Ps); 30: Dr-Termicin(Dr). Am: *A. mellifera*, Nv: *N. vitripennis*, Pt: *Protophormia terraenovae*, Sp: *Sarcophaga peregrine*, Dm: *D. melonagaster*, Hv: *Heliothis virescens*, Cn: *Centruroides noxius*, Cll: *C. limpidus limpidus*, Ps: *Pseudacanthotermes spiniger*, Dr: *Drepanotermes rubriceps*. Secondary structure elements (α-helix: cylinder; β-strand: arrow) and disulfide bridge connectivity are shown on the bottom of the alignment. Acidic residues in the propeptide are shown in pink and cleavage sites are boxed. Identical amino acids or constitutive replacements are shadowed in yellow and grey, respectively. Consensu/60% (Con.): - (negative), * (Ser/Thr), l (aliphatic), + (positive), t (tiny), a (aromatic), c (charged), s (small), p (polar), b (big), h (hydrophobic). Basic residues (K, R, H) are shown in blue. Residues split by phase 1 or 2 introns are boxed in green or purple. Omitted residues in carboxyl-termini are indicated by +aa.

In *A. mellifera*, there are only two defensins named defensin1 and defensin2 [33]. Amdefensin2 is a CITD with similar sequence, gene organization and molecular size to those from other insects. However, amdefensin1, also known as royalisin, has a lineage-specific carboxyl-terminal extension. Defensins with clear orthologous relationship with these two defensins have been identified in the *N. vitripennis *genome based on sequence similarity and identical precursor organization (signal peptide, propeptide and mature peptide) by our group [25]. These two defensins have undergone gene duplication to form a multigene family containing five members (navidefensin1-1, 1-2, 2-1, 2-2 and 2-3). A detailed analysis of their exon-intron organization allowed identifying *N. vitripennis*-specific intron loss and gain (Figure 1). For example, navidefensin1-1 has a phase 2 intron at the residue lysine following the CXC motif, which is conserved with amdefensin1, while navidefensin1-2 lacks this intron. 3'-intron loss or gain has been frequently observed in a large number of genes by a possible reverse transcription-mediated mechanism [34]. Whether dynamic evolution of introns is associated with the carboxyl-terminal extension in defensin1 remains an open question [35].

**Figure 2:**
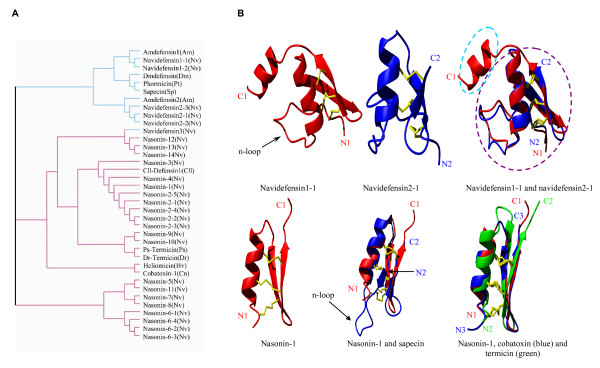
**Clustering and structure of DLPs**. **A**. Clustering analysis of the sequences in Figure 1 by the CLUSTAL program. Cyan and purple branches respectively represent CITDs, and nasonins and related peptides. Nasonin-2 and nasonin-6 are aligned as repeated domains; **B**. Structural models of DLPs from *N*.*vitripennis*. Navidefensin1-1 and navidefensin2-1 modeled *ab initio *by I-TASSER are shown in ribbon displayed by MolMol. Superimposition of these two structures shows an extra carboxyl-terminal domain in navidefensin1-1 structure. The model structure of nasonin-1 is compared with those of sapecin, termicin and cobatoxin.

Three-dimensional (3D) models of navidefensin1-1 and navidefensin2-1 were constructed by comparative modeling with sapecin's structure (PDB: 1L4V) [36] as a template, which confirmed their typical CSαβ folding, as identified by an α-helix and a two-stranded β-sheet. Residues spanning S^15^-S^23 ^in navidefensin1-1 and S^18^-A^26 ^in navidefensin2-1 form an α-helix with separate hydrophobic (A^16^, A^17 ^and L^22 ^in navidefensin1-1 or A^19^, A^21^, V^22^, L^25^, and A^26 ^in navidefensin2-1) and hydrophilic (S^15^, N^20^, and S^23 ^in navidefensin1-1 or S^18 ^and R^23 ^in navidefensin2-1) surfaces. Two β-stands (residues G^28 ^to C^31 ^and C^36 ^to R^39 ^in navidefensin1-1 or G^31 ^to C^34 ^and C^39 ^to R^42 ^in navidefensin2-1) constitute an antiparallel sheet with a γ-core region which has been considered as a functional determinant responsible for antimicrobial activity of some defensins [37]. Because of no suitable templates to build a model of the extended carboxyl-terminus of navidefensin1-1, alternatively an *ab initio *prediction method was employed. In both methods, a conserved CSαβ core was successfully predicted (Verify3D values > 0.2). For navidefensin1-1, the *ab initio *method predicted an α-helix in its carboxyl-terminus (residues K^44 ^to R^50^) (Figure 2B). The existence of an extra glycine in the carboxyl-terminus of navidefensin1-1 suggests that Phe^51 ^is likely amidated to provide a force for the helical stability, as predicted in bee defensin1 [33,38].

##### (2) Nasonins

In addition to the defensins mentioned above, we identified a new DLP subfamily without a propeptide, which contains fourteen members (named nasonin-1 to -14) (Figure 1). Sequence analysis revealed that nasonins are more closely related to two known non-classical defensins than CITDs in precursor organization, n-loop size and sequence similarity, one being antifungal termicin isolated from termites [39,40]; another being defensin1 from the Mexican scorpion *Centruroides limpidus limpidus *[41]. Intriguingly, such similarity was also observed between nasonins and a scorpion K^+ ^channel toxin cobatoxin [42,43] (Figure 1). These observations suggest that nasonins could have diverse functional features. For nasonin-2 and nasonin-6, their defensin unit has undergone 4 to 5 internal repeats, as described in fungal DLPs [32]. What's the potential function of such repeats and how these repeats occur will be further discussed below.

We also predicted the structure of nasonin-1 by comparative modeling using scorpion neurotoxin cobatoxin (PDB: 1PJV) [43] as a template. Although having a shorter n-loop than CITDs, nasonins can also adopt a typical CSαβ architecture (Figure 2B). The reliability of this model was evaluated by Verify3D with a score of 0.248. Overall, nasonin-1 more resembles structurally termicin (PDB: 1MM0) [44] and cobatoxin (Figure 2B), but they differ in molecular surface charge distribution (data not shown), which could explain functional diversity of these peptides, as observed between human beta-defensin2 and the snake toxin crotamine [45].

Navidefensin3 is a unique non-classical defensin because it has identical precursor organization and gene structure to navidefensin1 and 2 but similar n-loop size and amino acid sequence to nasonins. In fact, several non-classical insect defensins with a short n-loop were also found in Lepidoptera insects (e.g. gallerimycin, spodoptericin and the *B. mori *defensins) [46-48]. In the tree presented in Figure 2A, navidefensin3 is clustered together with nasonins rather than navidefensins, suggesting that this DLP is an evolutionary link between these two multigene subfamilies of defensins. Its lack in *A. mellifera *is consistent with the absence of the nasonin subfamily in this species.

##### (3) Navitricins

**Figure 3:**
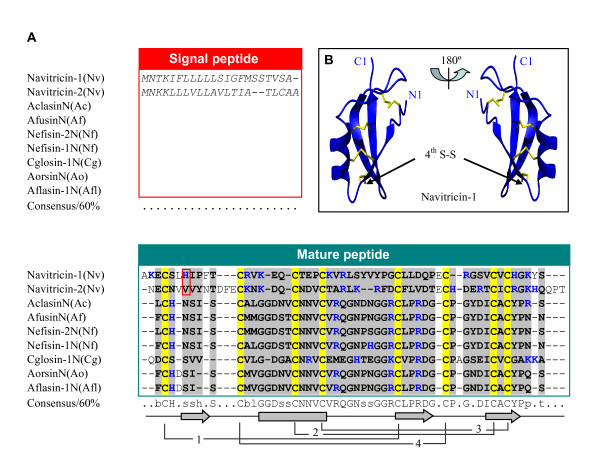
**Navitricins**. **A**. Sequence alignment of navitricins with fungal DLPs. The position of a phase 1 intron is boxed in red; **B**. The model structure of navitricin-1. Its fourth bridge, which links the n-loop to c-loop, is indicated by arrows. Ac: *Aspergillus clavatus*, Af: *A. fumigatus*, Nf: *Neosartorya fischeri*, Cg: *Chaetomium globosum*, Ao: *A. oryzae*, Afl: *A. flavus*.

We also found two unique DLPs (named navitricin-1 and -2) in the *N. vitripennis *genome, which possess 8 cysteines with an identical alignment pattern to the family III members of fungal DLPs [32]. Analysis of genomic sequences identified a phase 1 intron in the mature peptide-coding region of navitricins (Figure 3A).

As mentioned previously, navitricins have two additional cysteines which could form a fourth disulfide bridge. To confirm this, comparative modeling was again used to build the structure of navitricin-1 by choosing different templates, e.g. CITDs (sapecin and phormicin), the scorpion toxin BmTX1, and the plant sweet taste protein brazzein. In all these cases no acceptable models were generated and we thus tried to use the I-TASSER algorithm for structure simulation which produced a suitable model, as evaluated by Verify 3D (Figure 3B). Overall, this molecule is similar to the defensins described above including three identical disulfide bridges. Importantly, this model correctly predicts the fourth disulfide bridge linking the n- and c-loops together which makes the whole structure more compact. In fact, computational prediction was also applied to reveal an additional disulfide bridge in several insulin-like proteins [49].

##### (4) Biological activity

**Figure 4:**
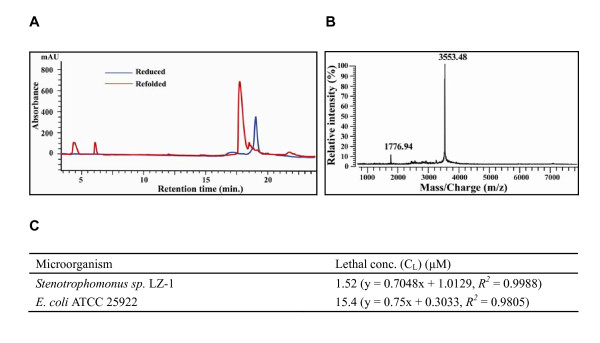
**Purification and characterization of nasonin-1**. **A**. RP-HPLC showing retention time difference between the reduced and refolded products; **B**. MALDI-TOF MS of the refolded nasonin-1; **C**. Antibacterial activities of nasonin-1.

Previous studies have highlighted a crucial role of α-helical and γ-core regions of insect defensins in inhibiting the growth of Gram-positive bacteria, whereas their n-loops could mediate antibacterial specificity [37,50,51]. Interestingly, all nasonins identified here have a shorter n-loop relative to CITDs. To confirm whether these novel DLPs have different antibacterial spectrum from CITDs, we chose nasonin-1 as a representative. Reduced nasonin-1 was chemically synthesized and its *in vitro *refolded was performed by using reduced and oxidized glutathione (GSH/GSSG) under an alkaline condition (pH = 8.0). Refolded peptide was further purified by reverse-phase high performance liquid chromatography (RP-HPLC) (Figure 4A). In comparison with the reduced form, refolded nasonin-1 has a slightly earlier retention time (18 min vs. 19 min). The molecular weight (MW) of refolded nasonin-1 detected by matrix-assisted laser desorption/ionization time of flight mass spectrum (MALDI-TOF MS) is 3553.48 Da, about 7 Da smaller than the reduced form (3560.8 Da), in favor of the formation of three disulfide bridges (Figure 4B).

Subsequently, we evaluated antibacterial and antifungal activities of nasonin-1 using classical inhibition zone assays [52] and found that nasonin-1 only displayed activity against two Gram-negative bacteria at micromolar concentrations with a lethal concentrations (C_L_) of 1.52 μM for *Stenotrophomonus sp*. LZ-1 and of 15.4 μM for *Escherichia coli *ATCC 25922 (Figure 4C). No effect was observed on Gram-positive bacteria and fungi. This is the first example describing a DLP mainly active on Gram-negative bacteria. Recombinant navidefensin2-2 was recently reported to be active on Gram-positive bacteria [25]. Whether such remarkable difference in target selectivity is associated with the change of n-loop length in these two *Nasonia *defensins needs further investigation.

#### AMPs rich in specific amino acids

AMPs rich in specific amino acids (e.g. glycine, proline, histidine, tryptophan or arginine) represent an additional class of AMPs acting on diverse microorganisms (e.g. bacteria, fungi, and virus) [53,54]. The majority of these molecules are unstructured and they often inhibit microbial growth by entering into cells and interacting with proteins involved in key metabolic processes [15]. Eighteen such AMPs have been identified here, including two known AMP families (abaecin and hymenoptaecin) [55,56].

##### (1) Abaecin

**Figure 5:**
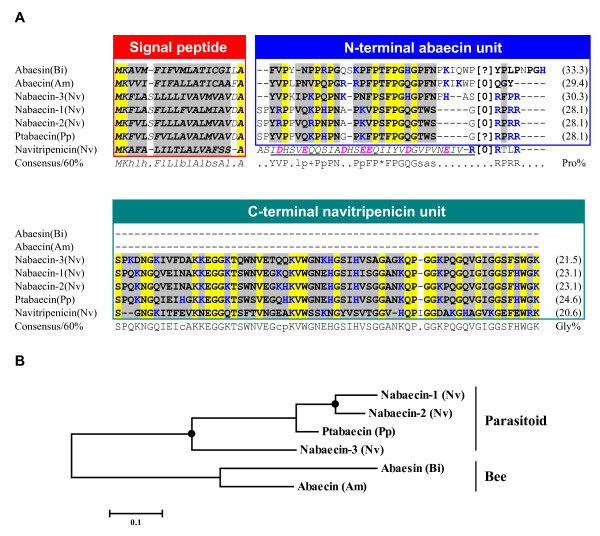
**Abaecin-like peptides**. **A. **Multiple sequence alignment. The propeptide sequence of navitripenicin is italized and underlined once. [0] indicates a phase 0 intron between the two residues and [?] represents unknown intron information due to the lack of genomic sequence. **B.** Phylogenetic tree constructed with the NJ method of MEGA 4.0. Solid circle represents gene duplication. Bi: *Bombus ignites*; Pp: *Pteromalus puparum*.

Abaecin is an inducible Pro-rich antibacterial peptide, firstly identified from the hemolymph of *A. mellifera *after bacterial challenge, and known to be regulated by the immune deficiency (IMD) pathway [55,57]. They can act on both Gram-positive and Gram-negative bacteria with a moderate inhibitory concentration when compared to apidaecin which was also from *A. mellifera *[55]. Subsequently, several abaecin-like genes were identified from the bee *A. cerana *and *Bombus ignites*, and the wasp *Pteromalus puparum *[58,59]. Searching for the *N. vitripennis *genome identified three genes encoding protein precursors with clear sequence similarity to abaecins in their amino-terminal part of mature peptides. We named these abaecin-like genes *nabaecin-1 *to *-3*. Analysis of these precursor sequences allowed recognizing a dibasic cleavage site (RR) for putative post-translational processing by which an amino-terminal part (called N-terminal abaecin unit, NtAU) corresponding to the bee abaecin will be released (Figure 5). The NtAU of nabaecin-3 shares the highest sequence identity (50%) with *A. mellifera *abaecin. To understand how nabaecins extended their carboxyl-termini, we further searched the *N. vitripennis *database and found that a peptide (named navitripenicin) possesses highly identical amino acid sequence to the carboxyl-termini of nabaecins (herein named C-terminal navitripenicin unit, CtNU), but the region corresponding to abaecin is replaced by an acidic propeptide with a dibasic cleavage site. High content of glycines together with positively charged characteristics suggest that CtNU and navitripenicin belong to putative AMPs. A conserved phase 0 intron located at a nearly identical position among all these peptides (Figure 5) supports their evolutionary relatedness.

##### (2) Hymenoptaecin

**Figure 6:**
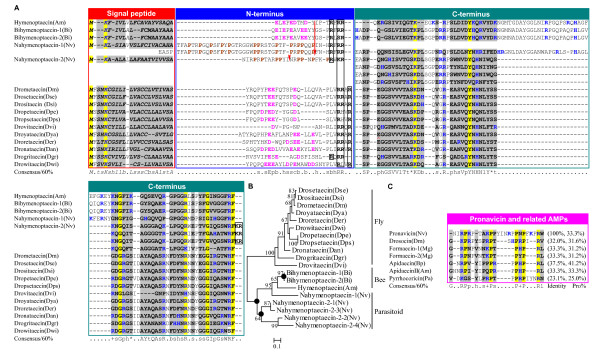
**Hymenoptaecin-like peptides**. **A**. Multiple sequence alignment. Red arrows label the position of phase 0 introns. A red "^" indicates a site in droyataecin that is corrected from TAG to GAG codon. Proline residues in amino-termini of nahymenoptaecins are bolded and shown in brown. Putative proprotein cleavage sites are boxed in black; **B**. Phylogenetic tree constructed with the NJ method of MEGA 4.0. Solid circle represents gene duplication; **C**. Comparison of pronavicin with known Pro-rich AMPs. Sequence identity with pronavicin and proline contents are shown here. Dse: *D. sechellia*, Dsi: *D. simulans*, Dpe: *D. persimilis*, Dps: *D. pseudoobscura*, Dvi: *D. virilis*, Dya: *D. yakuba*, Der: *D. erecta*, Dan: *D. ananassae*, Dgr: *D. grimshawi*, Dwi: *D. willistoni*, Mg: *Myrmecia gulosa*, Pa: *Pyrrhocoris apterus*, Bp: *Bombus pascuorum*.

Hymenoptaecin is an antibacterial peptide rich in glycine, which was identified only in Hymenopteran insects [56,59,60]. The mature hymenoptaecin is released by cleaving off a signal peptide and an acidic propeptide after bacterial infection. Expression of the *hymenoptaecin *gene was also regulated by the IMD pathway [57]. It inhibits the viability of Gram-negative and Gram-positive bacteria, including several human pathogens [56]. Similarity search of the *N. vitripennis *genome identified two hymenoptaecin-like peptides (named nahymenoptaecin-1 and nahymenoptaecin-2) with a unique amino-terminal AMP-like region corresponding to the propeptide of bee hymenoptaecins in position (Figure 6A). Functional characterization and evolutionary significance of nahymenoptaecin-1 has been described recently [26].

Nahymenoptaecin-2 presents a more interesting structural feature in the mature peptide region, whose amino- and carboxyl-termini are respectively a Pro-rich peptide (named pronavicin) and a hymenoptaecin-like peptide with four internal repeats. Overall, these repeats can be well aligned with hymenoptaecin except a deletion of 31 amino acids in each repeat. Similarly, a conserved phase 0 intron is also present in the amino-termini of bee and parasitoid hymenoptaecins, suggesting that these peptides diverged from a common ancestor after speciation.

Although hymenoptaecins are believed to be only present in Hymenoptera, our study reveals that similar peptides were also evolved by Dipteran *Drosophila *(Figure 6A). These fly-derived peptides display a chimeric characteristic because they have an acidic propeptides, as bee hymenoptaecins, but their mature peptides more resemble the repeat of nahymenoptaecin-2 in size. Moreover, *Drosophila *hymenoptaecins have lost their introns, consistent with the lineage-specific intron loss in these species [61]. All *Drosophila *hymenoptaecins clustering together in the tree (Figure 6B) indicates their monophyletic origin after separation of Diptera from Hymenoptera.

As a unique region of nahymenoptaecin-2, the amino-terminal pronavicin shares detectable sequence identity (25-41.2%) to other known Pro-rich AMPs from insects such as drosocin [62], apidaecin [12] and formaecin [63] (Figure 6C). Thus we synthesized this peptide to verify its antibacterial activity. Results showed that pronavicin was able to inhibit the growth of all Gram-positive and Gram-negative bacteria used here with differential potency. It displayed the strongest inhibitory effect on the Gram-positive bacterium *Bacillus megaterium *(*C*_*L *_= 3.11 μM) (Table 1). At high micromolar concentrations this peptide did not affect fungi and yeasts.

**Table 1:** Antibacterial activity of pronavicin.

Microorganism	Lethal conc. (C_L_) (μM)
*B. megaterium*	3.11 (y = 1.0867x + 1.019, *R*^2 ^= 0.998)
*M. luteus*	42.4 (y = 0.65x + 0.0175, *R*^2 ^= 0.9956)
*Bacillus sp.*	19.5 (y = 0.7125x - 0.2313, *R*^2 ^= 0.995)
*S. typhimurium*	15.8 (y = 0.3458x + 0.2525, *R*^2 ^= 0.9993)
*E. coli*	61.8 (y = 0.2375x + 0.0712, *R*^2 ^= 0.9774)
*Stenotrophomonus *sp. YC-1	78.0 (y = 0.3x + 0.0292, *R*^2 ^= 0.9959)
*Stenotrophomonus *sp. LZ-1	12.0 (y = 0.3625x + 0.3, *R*^2 ^= 0.9542)*

* indicates the semi-inhibition of pronavicin against *Stenotrophomonus. sp*. LZ-1.

#### Tachystatin-type AMPs

**Figure 7:**
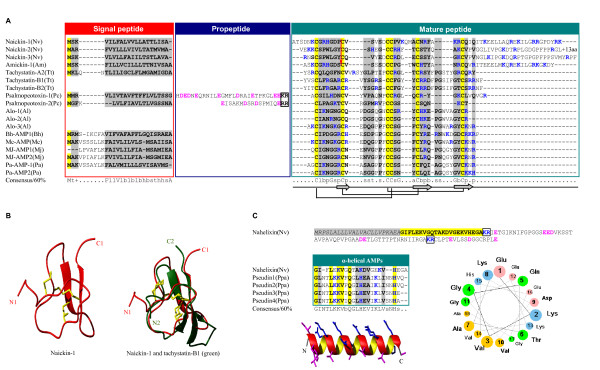
**Tachystatin-type and linear AMPs**. **A**. Multiple sequence alignment of tachystatin-type AMPs. A red arrow represents a phase 0 intron; **B**. Model structure of naickin-1 and its superimposition with tachystatin-B1; **C**. Nahelixin. Signal peptide and mature peptides are shaded in gray and yellow, respectively. In the alignment of nahelixin with known AMPs from frog, identical residues are bolded and shadowed in yellow while conservative substitutions are in gray. Structure modeled *ab initio *is displayed in ribbon with hydrophobic residues in pink and hydrophilic residues in blue. The helical wheel projection shows the amphiphilic characteristics of nahelixin. Putative proprotein cleavage sites are boxed in black. Tt: *Tachypleus tridentatus*, Pc: *Psalmopoeus cambridgei*, Al: *Acrocinus longimanus*, Bh: *Bradysia hygida*, Mc: *Mesembryanthemum crystallinum*, Mj: *Mirabilis jalapa*, Pa: *Phytolacca americana*, Ppa: *Pseudis paradoxa*.

ICK fold is an evolutionarily conserved structural motif shared by a large group of polypeptides with diverse sequences and bioactivities. For example, venomous animals (e.g. spider and scorpion) usually employed this fold to develop their neurotoxins affecting channels' functions [64,65] while horseshoe crabs, insects and some plants use ICK peptides as a part of their antibacterial defense system [66-69]. Peptides belonging to this fold type exhibit antibacterial (plant ICK peptides), antifungal (tachystatin and Alo-3), and chitin-binding (tachystatin-A) activities. Three peptides (named naickin-1, -2 and -3) with the ICK structure were characterized for the first time from the *N. vitripennis *genome that all share sequence similarity to an ICK peptide from *A. mellifera *(named amickin-1). All four peptides have a typical ICK cysteine arrangement pattern with a unique extended carboxyl-terminus rich in proline and positively charged residues. A phase 0 intron preceding the second cysteine is conserved between naickins and amickin-1 (Figure 7A), suggesting their homologous relationship. A typical cystine knot stabilized by three disulfide bridges can be observed in all these peptides, as revealed by comparative modeling using tachystatin-B1 [70] as a template. Figure 7B presents the model structure of naickin-1 (residues T^2 ^to T^40^) which is composed of an antiparallel β-sheet well fitted with tachystatin-B1.

#### Linear α-helical peptides

Linear peptides with an α-helical conformation are a large class of AMPs involved in immune response of arthropods, amphibians and vertebrates. Since cecropin, the first insect-derived linear AMP, was isolated from *Cecropia *moth [71], the number of such peptides dramatically increases in recent decades [72,73]. Several linear α-helical AMPs have also been characterized in social wasps [74-76]. From the *N. vitripennis *genome, we identified one AMP-like peptide (called nahelixin) belonging to this class (Figure 7C). The precursor of nahelixin is 118 residues in length with an amino-terminal signal peptide and a putative carboxyl-terminal propeptide. The predicted mature peptide of 22 amino acids shares about 40% sequence identity with four known antibacterial peptides from frogs [77]. Secondary structure prediction indicated it could adopt an α-helical structure (data not shown), which was further confirmed by *ab initio *structural prediction. An amphiphilic surface, with hydrophilic and hydrophobic residues separately arranged at two directions of the helical axis, is present in the model, as seen at the helical wheel projection (Figure 7C). Such structural feature represents a prerequisite for antibacterial activity of linear AMPs [6].

### Inducible expression profiles of *N. Vitripennis* AMP-like genes

**Figure 8:**
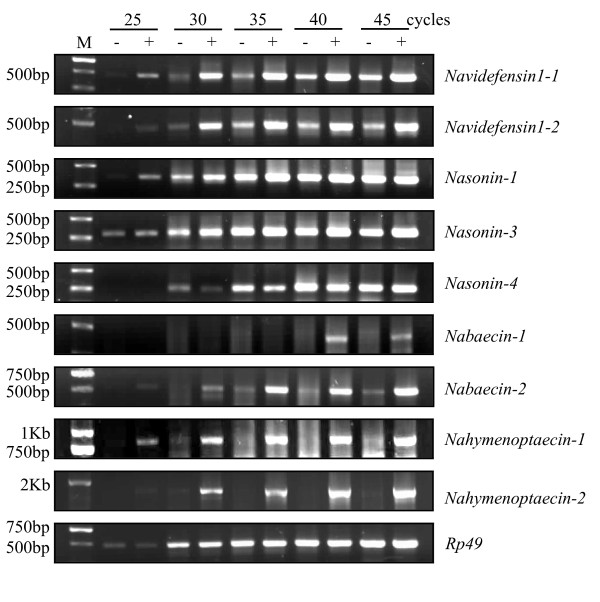
**Semi-quantitative RT-PCR detecting the inducible expression of AMP genes in *N. vitripennis *before and after bacterial infection**. M: DNA marker. -: non-challenged; +: challenged.

The up-regulation of AMP expression after infection is a common defense strategy by hosts to rapidly destroy invaders [5]. To evaluate whether *N. vitripennis *AMPs have such expression feature, we chose twelve genes that belong to 4 different categories (i.e. nabaecins, nahymenoptaecins, navidefensins, and nasonins) as representatives. By using RT-PCR and DNA sequencing, we found that six genes are constitutively expressed because their cDNAs can be amplified from both infected and non-infected *N. vitripennis *adults. These genes are respectively nasonin-1, nasonin-3, nasonin-4, nabaecin-2, together with navidefensin1-1 and 1-2, two recently cloned genes from the non-infected *N. vitripennis *adults [25]. Subsequently, we studied the expression profiles of all twelve genes after bacterial challenge by semi-quantitative RT-PCR. Results showed that only two genes (*nasonin-3 *and *nasonin-4*) whose expression was not induced by the bacteria. Four genes (*navidefensin1-1*, *1-2*, *nasonin-1 *and *nabaecin-2*) weakly expressed in the non-infected wasp were significantly up-regulated by bacterial challenge. Transcripts of *nabaecin-1*, *nahymenoptaecin-1 *and *nahymenoptaecin-2 *were only detected in the infected wasp, indicating that their expression is initiated by infection (Figure 8). Differential expression patterns among different AMPs may represent a combinational strategy to form a defense network against diverse microbial pathogens. Although three navidefensin2 genes were not detected to be expressed in the non-infected and infected adults, their defense role in different developmental stages of *Nasonia *is possible [25].

## Discussion

### AMPs in the parasitic wasp

With more and more genome sequences released, identifying new bioactive peptides/proteins by computational genomics approaches is becoming quite valuable. Some examples include hormones from nematode and mosquito, odorant binding-like proteins from honey bee, opossum immune genome, and reptile venom genes [78-82]. However, most strategies used are primarily based on sequence similarity that is useful in finding orthologues of known genes. An additional computational search strategy depends on the presence of conserved structural motifs within a peptide superfamily. Such examples include the identification of fungal DLPs and mammalian β-defensins [32,83]. Here, we identified the *N. vitripennis *AMPs in a genomic scale by using an integrated strategy which combines similarity search, pattern recognition and AMP characteristics (see additional file 1).

**Figure 9:**
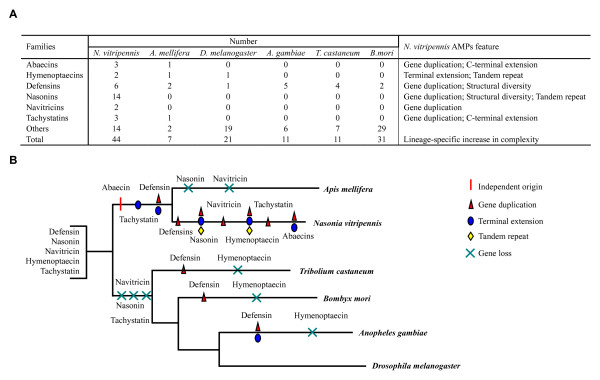
**AMPs from *N. vitripennis *and other insects**. **A**. Number and feature comparison; **B**. Evolutionary divergence of *N. vitripennis *AMPs, in which gene duplication, terminal extension, tandem repeat and gene loss events are highlighted.

**Figure 10:**
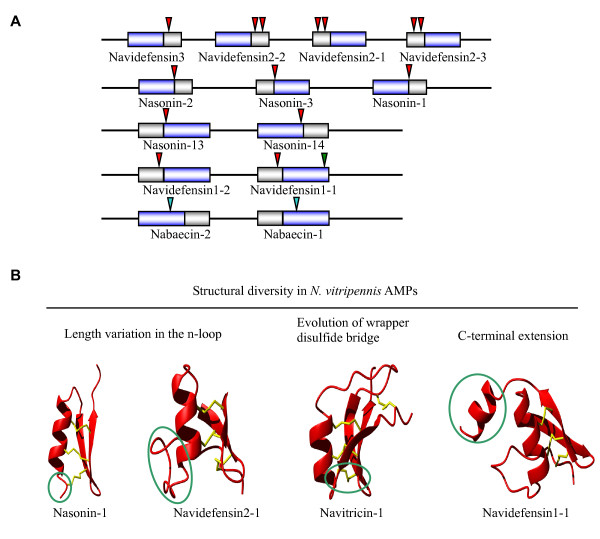
**Gene duplication and structural diversity of AMPs in *N. vitripennis***. **A**. Gene duplication. Chromosome fragments are shown in lines and genes coding AMPs are represented by box, in which gray one stands for signal and pro-peptide and blue one indicates mature peptide. Introns are indicated in triangles with phase 1 in red, phase 2 in green, and phase 0 in cyan; **B**. Structural diversity in *N. vitripennis *AMPs. Changes in structure relative to classical insect defensins are circled.

**Figure 11:**
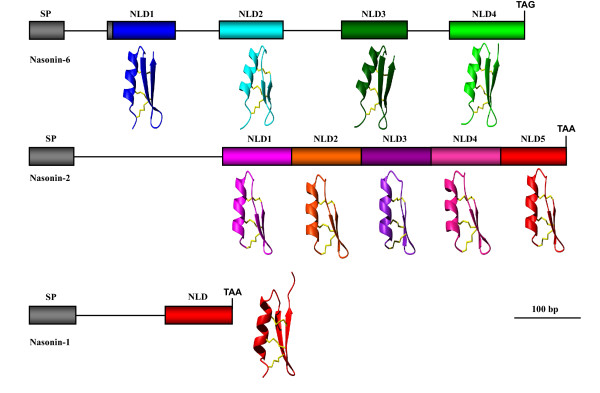
**Exon duplication of nasonins**. Peptides are indicated in boxes with color in gray for signal peptide (SP) and in other colors for NLDs. Lines mean the phase 1 introns between exons. Corresponding model structures of each domain are displayed in ribbon with different colors.

Our previous and current studies indicate that at least 44 AMPs are present in *N. vitripennis*. In comparison with *A. mellifera *AMPs [18,25,26], we found an obvious complexity increase of immune peptides in the parasitic wasp, both in number and protein precursor size (Figure 9). For example, *N. vitripennis *has three abaecins, six defensins and fourteen nasonins whereas *A. mellifera *has only 1 abaecin, 2 defensins and lacks nasonins. Besides changes in number, *N. vitripennis *also undergoes wide sequence extension. For instance, members in the nabaecin family from *N. vitripennis *have an extended carboxyl-terminus of about 65 residues relative to *A. mellifera *while nahymenoptaecins underwent duplication in their amino- or carboxyl-terminus (Figures 5 and 6, also see [26]) which largely enlarges their molecular size. In addition, the same type AMPs (e.g. defensins) also considerably change in several aspects: 1) The length variation in the n-loop (i.e. nasonins); 2) The development of a wrapper disulfide bridge [84] (i.e. navitricins); 3) Carboxyl-terminal extension (i.e. navidefensin1s) (Figure 10); and 4) For nasonin-2 and -6, the nasonin-like domain (NLD) repeats 4-5 times and thus represents another type of complexity increase (Figure 11).

In comparison with several non-Hymenopteran insects whose genome sequences have been released (e.g. *D. melanogaster *and *A. gambiae *from Diptera, *B. mori *from Lepidoptera, and *T. castaneum *from Coleoptera) [17-21], *N. vitripennis *still have evolved more AMP genes both in kinds and numbers (Figure 9A). Based on the phylogenetic relationship of holometabolous insects [85], we elucidate several evolutionary events (e.g. gene expansion, terminal extension and tandem repeat) which might have occurred along the parasitoid branch (Figure 9B) to result in the lineage-specific complexity increase in *N. vitripennis *AMPs for synergetic defense in parasitic condition [86].

### Evolution of the *N. Vitripennis* AMPs

Three different strategies have been recognized to shape the complex antimicrobial immune system in *N. vitripennis*:

#### Gene duplication

Adjacent chromosome location together with gene structure conservation strongly supports tandem gene duplication occurring in at least three AMPs in the *N. vitripennis *genome, including navidefensins, nabaecins and nasonins (Figure 10A). An emerging trend in the study of AMPs is that their evolution is often driven by Darwin positive selection following gene duplication [87]. However, we only detected weak positive selection signal in the amino-terminal abaecin unit (Table 2). No such signal was found in the gene families of defensins and nasonins, which is likely due to the sparse samplings of species used in this analysis because when more defensin sequences are included, accelerated amino acid substitutions were detected in the *Nasonia *lineage [25]. An additional change after gene duplication is structural diversification of *N. vitripennis *AMPs (Figure 10B). These structural variations may result in evolutionary novelty by changing structural basis related to function. For example, n-loop has been proven to be functionally important in mosquito defensin [50] and its change in size will probably influence the activity and antibacterial spectra of these defensins, as observed in navidefensin2-2 [25] and nasonin-1. There is also increasing awareness that alteration of the highly exposed wrapper disulfide bridge linkage pattern can lead to functional switch of scorpion sodium channel toxins via adjusting the conformation of key residues associated with toxin function [84]. In this regard, navitricins with such a disulfide bridge are examples for further research. Finally, a typical amphipathic surface is present in the carboxyl-terminal α-helical region of the navidefensin1 subfamily members, suggesting a new antimicrobial unit has evolved on the classical defensin scaffold.

**Table 2:** Maximum likelihood estimates of parameters and sites inferred to be under positive selection in the amino-terminal abaecin unit.

Model	*l*	LRT	Parameters	Positive selected sites
M0	-312.78		ω = 0.15	
M1a	-306.30		p_0 _= 0.80, ω_0 _= 0.046 p_1 _= 0.20, ω_1 _= 1.00	
M2a	-304.95	2.7	p_0 _= 0.80, ω_0 _= 0.05 p_1 _= 0.00, ω_1 _= 0.05315 p_2 _= 0.20, **ω_2 _= 5.91067**	4Y, 6P*, 8R, 11Q, 12K
M7	-306.58		p = 0.48 q = 1.62	
M8	-304.00	5.16	p_0 _= 0.80, p = 1.06, q = 14.00 (p_1 _= 0.20), **ω = 6.69**	4Y, 6P*, 8R, 11Q, 12K

Note: *l *is the log likelihood; LRT is a likelihood ratio test, which is twice the log likelihood difference (2Δ*l*) between null models (M1a and M7) and their alternative models (M2a and M8): M1a/M2a = 2.7 (χ^2 ^significant value: P < 0.3); M7/M8 = 5.16 (0.05 < P < 0.1). Positively selected sites identified by the BEB method under M2a and M8 with posterior probabilities (p) > 0.6 are shown, in which those with p > 0.95 are indicated by bold and *.

#### Exon duplication

In *N. vitripennis*, we identified two unusual defensin-like genes (i.e. nasonin-2 and nasonin-6) that encode proteins carrying 4-5 repeats of NLD without protease processing signals (Figure 11). Although we do not know how these genes originated, the gene structure of nasonin-6 gives a glimpse into its history. As shown in Figure 11, each NLD is encoded by a single exon, which is evidence for exon duplication [88]. From an evolutionary perspective, exon duplication mediated internal repeats could be viewed in the same light as gene duplication, which is usually considered to be the principal path for complexity increase of a protein. For example, the leucin-rich repeats (LRRs) in ribonuclease inhibitor (RI) are displayed in 1-30 tandem copies within a protein and such high LRR unit number could be critical for the evolution of both broad specificity and high affinity by RI [89]. The presence of multiple defensin repeats in nasonin-2 and -6 could have some structural significance if we consider the functional importance of forming oligomerization for membrane permeabilization of β-defensins [90]. Alternatively, new functional features were also evolved in some repeats, such as protease inhibitory activity [91] which may be useful in preventing from degradation of the whole molecule. The last domain of nasonin-2 has high sequence identity (86%) and a conserved phase 1 intron after signal peptide to nasonin-1, suggesting it originated from one ancestral copy of nasonin-1 by exon duplication and subsequent intron loss (Figure 1).

#### Exon-shuffling

**Figure 12:**
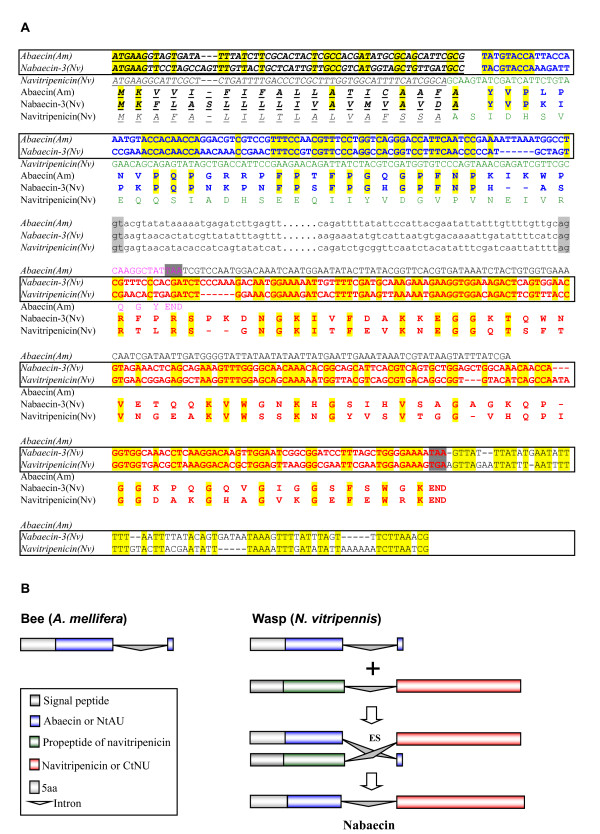
**A proposed exon shuffling mechanism for origin of nabaecin**. **A**. Sequence alignment of abaecin, nabaecin-3 and navitripenicin. Identical nucleotides and amino acids are shadowed in yellow and signal peptides are italized and underlined once. Alignable sequences are bolded and the corresponding nucleotides are boxed. Intron splice sites are showed in gray and omitted sequences are displayed as dots. Stop codons are shadowed in dark gray; **B**. The exon-shuffing model (for detailed description, see the text). Color codes are the same with those in Figure 12A.

Exon-shuffling represents a major mechanism by which a limited set of modules can be shuffled by intronic recombination. Such mechanism has significantly contributed to the emergence of novel complex multidomain proteins, novel functions and increased organismic complexity. Gene structure conservation in multidomain immune molecules suggests that exon shuffling may be involved in the evolution of the immune system [92]. However, in most case tracing such event is rather difficult due to accelerated sequence changes. Nabaecins, chimeric genes of abaecin and navitripenicin as mentioned previously (Figure 5), provides some clues to see a history of immune-related genes, in which exon-shuffling is likely involved. At the sequence level, the NtAU of nabaecin-3 is highly similar to *A. mellifera *abaecin (41.2% (protein) or 45.1% (DNA)), while its CtNU shares 41.4% (protein) and 56.2% (DNA) identity to navitripenicin. And the sequence identity extends to their 3' UTRs (Figure 12A). At the genomic level, all these three genes retain a conserved phase 0 intron at identical positions (Figure 5), indicating that they are evolutionarily related. In all these genes, CtNU and navitripenicin are encoded by a single exon. Therefore, we assume that after the wasp diverged from the bee lineage, an exon-shuffling, mediated by the conserved phase 0 intron, occurred between the ancestral ortholog of the *A. mellifera *abaecin and a duplicated navitripenicin gene, which resulted in the emergence of the first nabaecin gene. In subsequent evolution, a multigene family of three members formed by gene duplication (Figure 12B).

## Conclusions

By using a combined computational and experimental strategy, we established for the first time the *N. vitripennis *peptidome associated with innate immunity. Three basic evolutionary scenarios are recognized in the generation of a complex antimicrobial system in *N. vitripennis*. Our work presented here will offer a basic platform for further studying the immunological and evolutionary significances of these newly discovered AMPs in parasitic insects. Whether these AMP-like genes with a complicate structure or much more copies are involved in parasitism remains an open question which constitutes our next research direction. In particular, our efficient approaches for finding new AMPs in a genomic scale can easily be applied to other model organisms including humans, which will accelerate our understandings on AMP-mediated innate immunity at a defense network level. Further functional characterization of these parasitic wasp-derived AMPs will also help explore new-type of drug leads for anti-infective therapy.

## Methods

### Database searches

Strategies for gene discovery used here are provided in the supplementary information (see additional file 1). BLASTP and TBLASTN programs were used to characterize orthologues of known AMPs from *A. mellifera *against the database of *N. vitripennis *[93]. When searching for hymenoptaecin in flies, BLAST was carried out in Flybase [94]. The program WinGene [95] was employed to predict and translate a complete open reading frame from a selected nucleotide sequence. The *N. vitripennis *protein sequences downloaded from GenBank [96] and the FTP site [97] were applied to perform ScanProsite on the ExPaSy sever [98].

### Characteristics identification

All sequences we identified as AMP-like peptides were submitted to SignalP 3.0 server [99] for signal peptide prediction. Peptide characteristics identified here were further analyzed on the ExPaSy server [100] for estimation of molecular weights and calculation of net charges at Protein Calculator [101]. Secondary structure prediction was done on the ExPaSy server [100]. To predict amphiphilic structure of peptides, helical wheels models were calculated on the server [102].

### Sequence alignment and structural analysis

Amino acid sequences were aligned using Clustal X [103] with fine adjustment by hands and phylogenetic trees were constructed by MEGA 4.0 [104]. Structures were modeled from *ab initio *by I-TASSER on-line [105-107] or on the Structural Bioinformatics server by comparative modeling method [108]. Structure evaluation was carried out by Verify 3D [109,110]. The MultiProt server was used to do structural superimposition and calculation of root mean square deviation (RMSD) [111]. All model structures predicted here have been despoited in Protein Model DataBase [112] under id number of PM0075686-PM0075709.

### Maximum likelihood analysis

Codon-substitution models were selected to estimate the nonsynonymous-to-synonymous rate ratio (ω = dN/dS) using the CODEML program of the PAML software package [113]. Four models recommended by Yang make two likelihood ratio tests (LRTs) by M1a/M2a and M7/M8: M1a (nearly neutral model) constraints a proportion p_0 _of conserved sites with 0 < ω < 1, while a proportion p_1 _= 1-p_0 _of neutral sites with ω_1 _= 1; M2a (positive selection model) adds an extra class of sites with the proportion p_2 _= 1-p_0 _-p_1 _and with ω estimated from the data. M7 (β distribution model) does not allow for positively selected sites and M8(β and ω model) adds an extra class of sites to M7, allowing for ω > 1, which means the presence of positively selected sites. In addition, Model 0 (M0) assuming one ω for all sites that does not allow the existence of positive selection was chosen for negative control [114,115]. Upon detection of the positively selected signals, the calculation of posterior probabilities was completed using the Bayes Empirical Bayes (BEB) method [115,116].

### Chemical synthesis and oxidative refolding

Pronavicin and reduced nasonin-1 were chemically synthesized by Xi'an Huachen Bio-Technology Co., Ltd. (Xi'an, China). The cyclization reaction to form disulfide bridges in the reduced nasonin-1 molecule was carried out in 0.1 M Tris-HCl (pH = 8.0) with 2 mM GSH and 0.2 mM GSSG. Refolded peptide was purified to be homogeneity by C18-RP-HPLC. Its MW was determined by MALDI-TOF MS on a Kratos PC Axima CFR plus (Shimazu Co. LTD, Kyoto).

### Antimicrobial assays

Inhibition zone assays were carried out to evaluate antimicrobial activities of peptides [28,52,117]. Briefly, 50 μl bacteria or fungal spores with OD_600 _= 0.5 were inoculated to pre-heated 6 ml Luria-Bertaini's medium (1% bactotryptone, 0.5% bactoyeast extract and 0.5% NaCl) for bacteria or MEA medium (1% Malt Extract, 0.1% Peptone, 2% Glucose) for fungi containing 0.8% agar. The mixture was then spread on a 9-cm Petri dish, giving a depth of 1 mm. After settling, 2-mm wells were punched in the plate and then 2 μl of peptide under different concentrations was added to each well. Diameters of the inhibition zones were measured 16 hours after incubation at 30°C. *C*_*L *_was calculated by the Hultmark's method [52]. The microorganisms used in this assay were listed in additional file 3.

### Establishment of the infection model

*N. vitripennis *was maintained as a laboratory culture on pupae of the housefly *Musca domestica*. They were reared under a 14:10 light-dark cycle at 25°C in glass containers fed on 20% (v/v) honey solution. A standard method was applied to establish an infection model [118] of *N. vitripennis*. Briefly, a bacterial mixture of *E. coli *ATCC 25922 and *M. luteus *which had been separately incubated overnight was injected into abdomen of *N. vitripennis *adults with a micro-injector. About 50 wasps were challenged in total. Non-injected wasps were used as a control.

### Preparation of total RNA

For isolating *N. vitripennis *total RNA, 50 wasps challenged for 16 hours by bacteria were grounded into fine powder in liquid nitrogen. The Trizol reagent (SBS Genetech, Beijing) was used to prepare total RNA according to the supplier's instructions. This method was also applied to prepare non-challenged total RNA [25].

### cDNA Cloning

Reverse transcription of total RNAs prepared from non-infected or infected *N. vitripennis *was performed using RT PreMix kit (TransGen, Beijing) and a universal oligo (dT) - containing adaptor primer dT3AP. The first-strand cDNA amplification in both non-infected and infected wasps was performed by using an AMP-like gene-specific primer and the universal primer 3AP according to the method described [119]. PCR product was cloned into pGEM-T (TIANGEN, Beijing), and transformed into *E. coli *DH5α competent cells (TransGen, Beijing). Their sequences were confirmed by DNA sequencing. Primers used in this study are all listed in additional file 4.

### Semi-quantitative RT-PCR

For semi-quantitative RT-PCR, an AMP-like gene were amplified from non-infected or infected cDNAs by using a gene-specific primer and 3AP and PCR products obtained from different cycles (i.e. 25, 30, 35, 40, 45) were taken for comparison of the amounts of these products by electrophoresis on 1.5% agarose gels. *N. vitripennis *ribosome protein RP49 was chosen as an internal control which was amplified by using primers NvRp49-F2/3AP and the same cDNA templates.

## Competing interests

The authors declare that they have no competing interests.

## Authors' contributions

SZ designed the experiments. CT, SZ and GY wrote the paper. CT conducted database search. CT, BG and QF performed the experiments. All authors read and approved the final manuscript.

## Supplementary Material

Additional file 1 **Strategy of database searches of putative *Nasonia vitripennis *antimicrobial peptides**. A diagram for the computational identification of putative AMPs. Click here for file

Additional file 2 **Summary of *N. vitripennis *antimicrobial peptides**. A table for the summary of *N. vitripennis *antimicrobial peptides. Click here for file

Additional file 3 **Microorganism sources used in this work**. A table for the microorganisms used here. Click here for file

Additional file 4 **The PCR primers used in this work**. A table for primers used here. Click here for file
