# Supplementary material for: Antimicrobial peptide-like genes in Nasonia vitripennis: a genomic perspective
Source: BMC Genomics. 2010 Mar 19;11:187. doi: 10.1186/1471-2164-11-187 (PMC2853521; doi:10.1186/1471-2164-11-187)
Supplement: Additional file 1 — Strategy of database searches of putative Nasonia vitripennis antimicrobial peptides. A diagram for the computational identification of putative AMPs. [file 1471-2164-11-187-S1.DOC]

**Additional file 1**

Strategy of database searches of putative *Nasonia vitripennis* antimicrobial peptides.

***Apis mellifera* known antimicrobial peptides**

***BLASTP***

***TBLASTN***

***Nasonia vitripennis* protein and genomic database**

**(**[**http://www.ncbi.nlm.nih.gov**](http://www.ncbi.nlm.nih.gov/)**)**

***Filter using signal peptide***

**Putative *Nasonia vitripennis* antimicrobial peptides**

***Pattern recognition***

***Nasonia vitripennis* proteins in GenBank**

**(**[**http://www.ncbi.nlm.nih.gov**](http://www.ncbi.nlm.nih.gov/)**)**

***Pattern search***

**(**[**www.expasy.org**](http://www.expasy.org/)**)**

**Putative *Nasonia vitripennis* antimicrobial peptides**

***Filter with signal peptide and***

***characteristics of known***

***antimicrobial peptides***

***Nasonia vitripennis* proteins in GenBank**

**(**[**http://www.ncbi.nlm.nih.gov**](http://www.ncbi.nlm.nih.gov/)**)**
