# Supplementary material for: Antimicrobial peptide-like genes in Nasonia vitripennis: a genomic perspective
Source: BMC Genomics. 2010 Mar 19;11:187. doi: 10.1186/1471-2164-11-187 (PMC2853521; doi:10.1186/1471-2164-11-187)
Supplement: Additional file 2 — Summary of N. vitripennis antimicrobial peptides. A table for the summary of N. vitripennis antimicrobial peptides. [file 1471-2164-11-187-S2.DOC]

***Additional file2*** *Summary of N. vitripennis antimicrobial peptides.*

| Name | GenBank No. | EST | Tpye | Size | | MW(Da) | Net Charge |
| --- | --- | --- | --- | --- | --- | --- | --- |
|  |  |  |  | ORF | MP |  |  |
| Nabaecin-1^*^ | XP_001600519 | EV431137 | PG-rich | 117 | 97 | 10395.62 | +11.9 |
| Nabaecin-2^*^ | AAZX01012011 | ES633264 | PG-rich | 117 | 97 | 10381.56 | +11.9 |
| Nabaecin-3 | AAZX01005518 |  | PG-rich | 118 | 98 | 10538.89 | +11.9 |
| Navitripenicin | AAZX01007139 |  | G-rich | 118 | 63 | 6978.72 | +3.4 |
| Nahymenoptaecin-1^*^ | XP_001607881 | ES633234 | G-rich | 209 | 94 | 10164.21 | +3.1 |
| Nahymenoptaecin-2^a*^ | XP_001602522 |  | PG-rich | 323 | 24 | 2941.44 | +5.9 |
| Navidefensin1-1 | XP_001603321 | ES645088 | CSαβ | 101 | 52 | 5359.12 | -0.3 |
| Navidefensin1-2 | AAZX01000740 | GE450320 | CSαβ | 101 | 52 | 5378.22 | +5.0 |
| Navidefensin2-1 | XP_001605258 |  | CSαβ | 106 | 42 | 4657.49 | +6.0 |
| Navidefensin2-2 | XP_001605277 |  | CSαβ | 118 | 42 | 4640.40 | +6.0 |
| Navidefensin2-3 | XP_001605239 |  | CSαβ | 103 | 43 | 4629.17 | +1.0 |
| Navidefensin3 | AAZX01001916 |  | CSαβ | 87 | 34 | 3570.22 | +4.7 |
| Nasonin-1^*^ | XP_001607888 | GE380814 | CSαβ | 56 | 33 | 3555.01 | +1.7 |
| Nasonin-2^b^ | AAZX01000698 | GE397561 | CSαβ | 195 |  |  |  |
| Nasonin-3^*^ | XP_001607885 | GE397057 | CSαβ | 63 | 41 | 4662.54 | +3.0 |
| Nasonin-4^*^ | AAZX01010653 | GE361410 | CSαβ | 58 | 39 | 4390.05 | +2.0 |
| Nasonin-5 | XP_001605675 | GE384375 | CSαβ | 60 | 38 | 4065.71 | +1.0 |
| Nasonin-6^b^ | XP_001606119 | ES633199 | CSαβ | 153 |  |  |  |
| Nasonin-7 | XP_001599338 | ES651177 | CSαβ | 61 | 40 | 4620.22 | +1.7 |
| Nasonin-8 | XP_001608211 | ES641264 | CSαβ | 83 | 63 | 7065.89 | -2.8 |
| Nasonin-9 | XP_001607446 | GE416153 | CSαβ | 63 | 46 | 4847.70 | +1.7 |
| Nasonin-10 | AAZX01000070 |  | CSαβ | 64 | 39 | 4393.13 | +0.7 |
| Nasonin-11 | XP_001599336 | GE386279 | CSαβ | 73 | 54 | 5911.69 | -0.3 |
| Nasonin-12 | AAZX01000545 |  | CSαβ | 71 | 52 | 5804.70 | +2.0 |
| Nasonin-13 | XP_001602681 |  | CSαβ | 66 | 46 | 5240.94 | +4.0 |
| Nasonin-14 | XP_001605239 | GE356569 | CSαβ | 67 | 48 | 5522.34 | +1.2 |
| Navitricin-1 | XP_001607644 |  | CSαβ | 74 | 52 | 5857.87 | +2.1 |
| Navitricin-2 | XP_001607635 |  | CSαβ | 77 | 56 | 6579.38 | -1.8 |
| Naickin-1 | XP_001600083 | GE460590 | ICK | 81 | 61 | 6903.99 | +6.0 |
| Naickin-2 | XP_001599718 | GE460136 | ICK | 90 | 70 | 7659.84 | +2.5 |
| Naickin-3 | AAZX01007976 | GE361308 | ICK | 80 | 60 | 6665.65 | +1.7 |
| Glynavicin-1 | XP_001602829 |  | G-rich | 109 | 42 | 4667.96 | +3.1 |
| Glynavicin-2 | XP_001601014 |  | G-rich | 149 | 130 | 11743.70 | +0.2 |
| Glynavicin-3 | XP_001604172 |  | G-rich | 72 | 54 | 5447.85 | +2.9 |
| Glynavicin-4 | XP_001600869 |  | G-rich | 149 | 91 | 9386.34 | +7.9 |
| Glynavicin-5 | XP_001607541 |  | G-rich | 135 | 81 | 9052.61 | +4.5 |
| Glynavicin-6 | XP_001607559 | ES639803 | G-rich | 127 | 96 | 9563.56 | +3.3 |
| Glynavicin-7 | XP_001600805 | ES644269 | G-rich | 140 | 97 | 9176.29 | +7.9 |
| Hisnavicin-1 | XP_001606555 | EV427937 | HP-rich | 93 | 77 | 9107.21 | +2.7 |
| Hisnavicin-2 | XP_001606568 | ES636083 | H-rich | 130 | 114 | 13510.98 | +4.4 |
| Hisnavicin-3 | XP_001607730 |  | HP-rich | 139 | 101 | 11314.17 | +0.3 |
| Hisnavicin-4 | XP_001607139 | ES647246 | H-rich | 144 | 123 | 14173.44 | +0.7 |
| Hisnavicin-5 | XP_001599590 | ES648871 | H-rich | 105 | 86 | 9991.44 | +4.9 |
| Nahelixin | XP_001607338 | EV427656 | α-helix | 118 | 22 | 2312.61 | -0.8 |

Note: ^a^ the features of the amino-terminal pronavicin. ^b^ the characteristics not determined due to tandem repeats. ^*^ evidence for transcription is further provided by RT-PCR and cDNA cloning in this work.
