# Supplementary material for: Antimicrobial peptide-like genes in Nasonia vitripennis: a genomic perspective
Source: BMC Genomics. 2010 Mar 19;11:187. doi: 10.1186/1471-2164-11-187 (PMC2853521; doi:10.1186/1471-2164-11-187)
Supplement: Additional file 3 — Microorganism sources used in this work. A table for the microorganisms used here. [file 1471-2164-11-187-S3.DOC]

**Additional file 3** Microorganism sources used in this work.

| Microorganisms | Source |
| --- | --- |
| **Fungi**  *Neurospora crassa*  *Neurospora crassa* MUT16  *Aspergillus fumigatus* CCTCC AF93024  *Beauveria* sp. CCTCC AF 93313  *Geotrichum candidum* CCTCC AY 93038 | Center for Microbial Resources, Institute of Microbiology, Beijing, China  Gift from Dr. Karin Thevissen, Leuven University, Belgium  China Center for Type Culture Collection, Wuhan, China  China Center for Type Culture Collection, Wuhan, China  China Center for Type Culture Collection, Wuhan, China |
| **Yeasts**  *Saccharomyces cerevisiae* CCTCC AY 92003  *Candida albicans* | China Center for Type Culture Collection, Wuhan, China  Gift from Prof. Fengyan Bai, Institute of Microbiology, Beijing, China |
| **Gram-positive bacteria**  *Bacillus megaterium* CCTCC AB 91020  *Micrococcus luteus*  *Bacillus* sp.DM-1 | China Center for Type Culture Collection, Wuhan,  China Center for Microbial Resources, Institute of Microbiology, Beijing, China  Gift from Prof. Chuanling Qiao, Institute of Zoology, Beijing, China |
| **Gram-negative bacteria**  *Agrobacterium tumefaciens* CCTCC AB 92026  *Pseudomonas aeruginosa* CCTCC AB 91095  *Salmonella typhimurium* CCTCC AB 94007  *Serratia marcescens* CCTCC AB90025  *Escherichia coli* ATCC 25922  *Shewanella Oneidensis* MR-1  *Stenotrophomonus* sp. YC-1  *Stenotrophomonus* sp. LZ-1  *Klebsiella* sp. F51-1-2  *Pseudomonus putida* | China Center for Type Culture Collection, Wuhan, China  China Center for Type Culture Collection, Wuhan, China  China Center for Type Culture Collection, Wuhan, China  China Center for Type Culture Collection, Wuhan, China  Center for Microbial Resources, Institute of Microbiology, Beijing, China  Gift from Dr. Jianguo Zhou, Oak Ridge National Laboratory, Oak Ridge, TN, USA  Gift from Prof. Chuanling Qiao, Institute of Zoology, Beijing, China  Gift from Prof. Chuanling Qiao, Institute of Zoology, Beijing, China  Gift from Prof. Chuanling Qiao, Institute of Zoology, Beijing, China  Center for Microbial Resources, Institute of Microbiology, Beijing, China |
