# Supplementary material for: Antimicrobial peptide-like genes in Nasonia vitripennis: a genomic perspective
Source: BMC Genomics. 2010 Mar 19;11:187. doi: 10.1186/1471-2164-11-187 (PMC2853521; doi:10.1186/1471-2164-11-187)
Supplement: Additional file 4 — The PCR primers used in this work. A table for primers used here. [file 1471-2164-11-187-S4.DOC]

**Additional file 4** The PCR primers used in this work

| Name | Sequences (5’ to 3’) |
| --- | --- |
| dT3AP | CTGATCTAGAGGTACCGGATCCTTTTTTTTTTTTTTTTT |
| 3AP | CTGATCTAGAGGTACCGGATCC |
| NvRp49-F2 | GGACGAAGAAGTTCATCAGG |
| Navidef1-1F | ATGAAGCTCCTACTCGTTGT |
| Navidef1-2F | ATGAAATTCCTGATTATTGCT |
| Navidef2-1F | ATGAAGGTCCTCGTGGCTCTC |
| Navidef2-2F | ATGAAGGTCCTCGTTGTTTTG |
| Navidef2-3F | ATGAAGTTCCTGACGGTTTTC |
| Nasonin-1/2FP | CATGAAGGCTGTATACTT |
| Nasonin-3 | ATGGATCCGATGACGATGACAAGTCTGCTATTCCACACTGCAGCCCG |
| Nasonin-4 | ATGGATCCGATGACGATGACAAGGAGTCAGCTTACTGCGGAAGACTC |
| Nabaecin-1/3FP | ATGAAGTTCCTMGCMAGTTT |
| Nabaecin-2FP | ATGAAGTTCGTCCTCAGTTT |
| Nahym-F1 | ATGAAGCTTTCGATTGCTGT |
| Nahymenoptaecin-2FP | ATGAAAGCAGCCCTCGCA C |
